# Palmar Trans-scaphoid Perilunar Dislocation: A Case Report and a Literature Review of Clinicodemographic Characteristics and Management Options

**DOI:** 10.7759/cureus.51599

**Published:** 2024-01-03

**Authors:** Roy Safi, Maher Ghandour, Antoine Azzi

**Affiliations:** 1 Orthopedic Surgery Department, Faculty of Medical Science, Lebanese University, Beirut, LBN; 2 Orthopedic Department, CHU Grenoble Alpes, Grenoble, FRA; 3 Orthopedic Department, Centre Hospitalier Universitaire (CHU) - Hopital Libanais Geitaoui, Lebanese University, Beirut, LBN

**Keywords:** literature review, internal fixation, open reduction, case report, trans-scaphoid perilunar dislocation

## Abstract

Palmar trans-scaphoid perilunar dislocation is a rare finding in clinical practice. Herein, we report a case of a young right-handed male hairdresser brought to the emergency room after a heavy blow to his right hand. An X-ray showed a palmar dislocation of the capitate, carpus, and distal scaphoid relative to the lunate, which remains in normal alignment with the radius, with the proximal scaphoid maintaining attachment to the lunate. Open reduction and internal fixation were done in the first week after trauma.

## Introduction

Palmar trans-scaphoid perilunar dislocation is rarely observed in clinical practice; however, it is associated with several diagnostic and treatment challenges. Usually results from high-energy injuries on the dorsum of the wrist. This is directly opposite to the mechanism that produces a dorsal perilunar dislocation [1]. The fracture line passes through the scaphoid and induces rupture of the lunotriquetral and lunocapitate ligaments, otherwise, the dorsal scapholunate ligament remains intact [2]. According to Mayfield classification, carpal instability has been divided into four stages: stage 1, scapholunate dissociation with disruption of scapholunate ligament; stage 2, with additional disruption capitolunate ligament; stage 3, perilunate dislocation with additional disruption of lunotriquetral ligament and lunate stays in position while carpus dislocates; and stage 4, lunate dislocation with additional disruption of dorsal radiocarpal ligament and lunate forced volar or dorsal while carpus remains aligned [3]. The diagnosis of palmar trans-scaphoid perilunar dislocation can be reached through proper history, proper physical exam, and radiological assessment through X-rays and computed tomography (CT). Herein, we report a case of a young male with right wrist trauma.

## Case presentation

History and presentation

A 37-year-old male patient was brought to the emergency room after a heavy, posteroanterior blow to the dorsum of his right hand against a wall. The patient complained of severe pain and swelling in the right wrist. Informed consent was taken from the patient before the conduct of this research. The patient had no prior medical or surgical history. A physical exam of the right wrist was conducted, revealing extensive swelling over the right wrist, tenderness was evident. No evidence of median nerve compression.

Radiographical assessment

**Figure 1:**
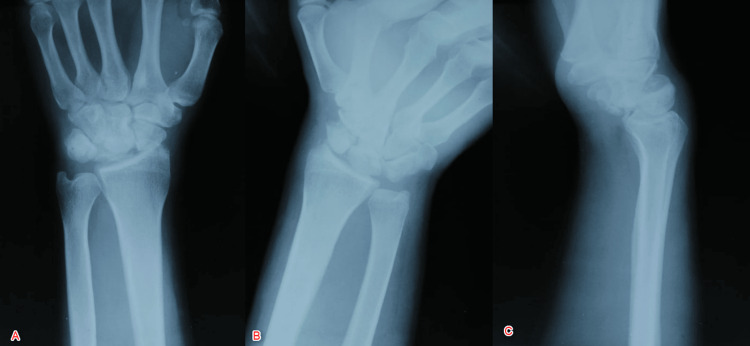
X-ray of the right wrist showing palmar dislocation of the capitate and distal scaphoid relative to the lunate A: Anteroposterior view; B: Oblique/Scaphoid view; C: Lateral view

An X-ray of the right wrist (anteroposterior, scaphoid, and lateral view) revealed a palmar dislocation of the capitate, carpus, and distal scaphoid relative to the lunate, which remains in normal alignment with the radius, and the proximal scaphoid maintains its relationship with lunate (Figure 1).

**Figure 2:**
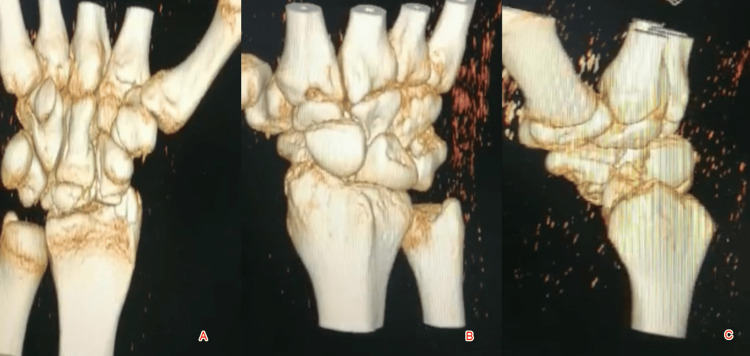
A CT scan of the right wrist with 3D reconstruction showing comminution of the distal scaphoid and the volar part of the lunate and displaced fracture of the tip of the radial styloid A: Anteroposterior view; B: Oblique/Scaphoid view; C: Lateral view

CT scan with 3D reconstruction of the wrist revealed the same result plus comminution of the distal scaphoid and the volar part of the lunate and undisplaced fracture of the tip of the radial styloid (Figure 2).

Management

**Figure 3:**
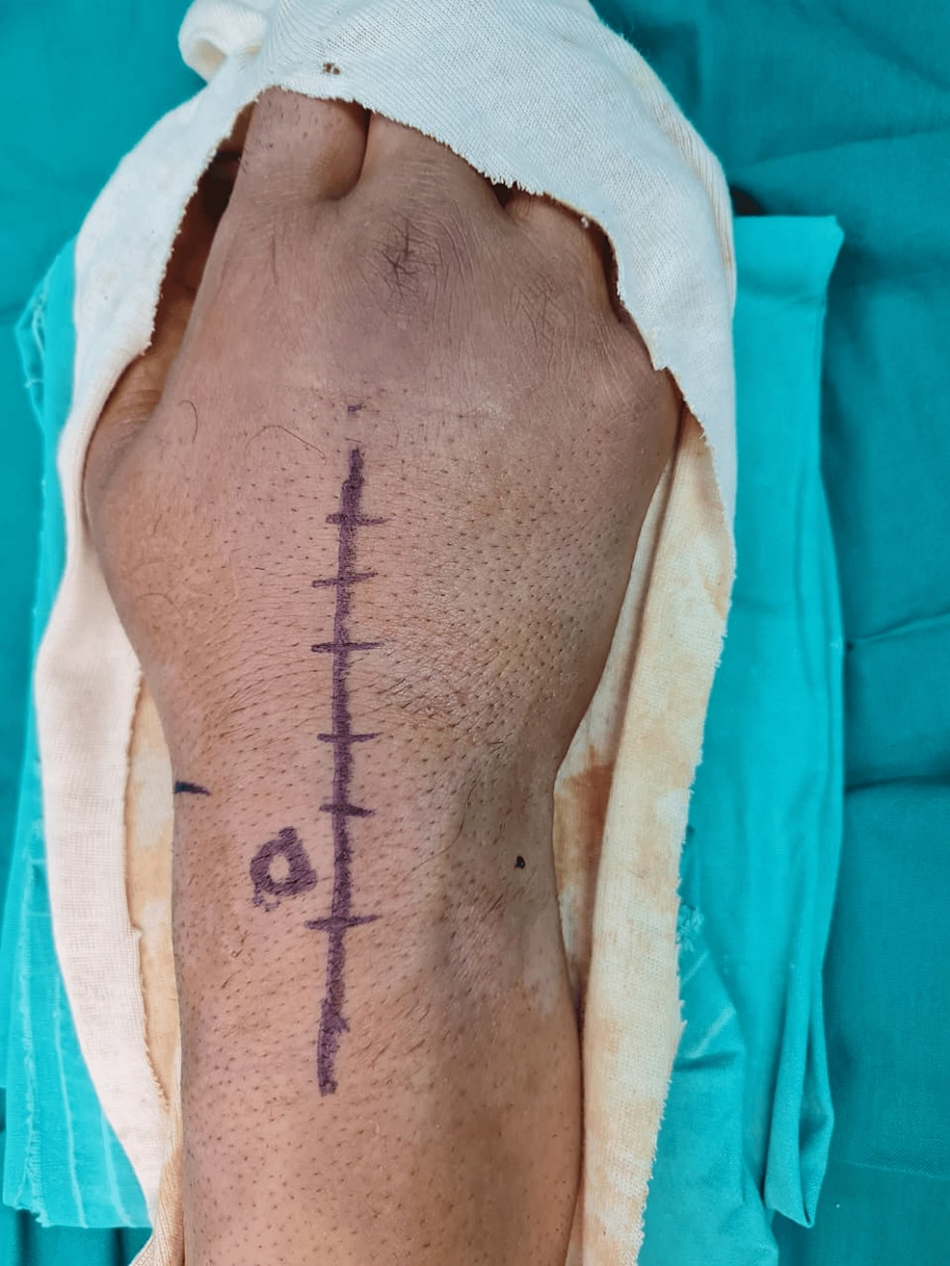
Open-reduction/internal fixation of the right wrist of the patient through the dorsal approach (medially to Lister’s tubercle)

**Figure 4:**
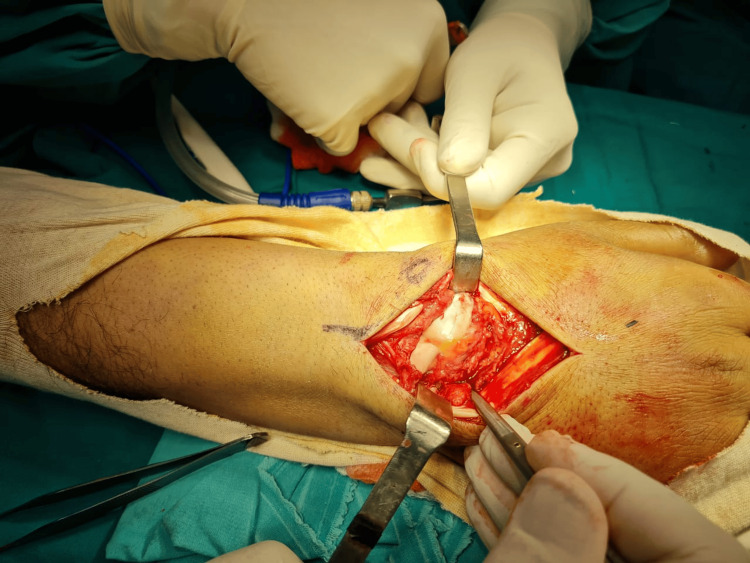
Radially-based, v-shaped capsulotomy (the incision of the extensor retinaculum was done between the third and fourth compartments)

Following a thorough assessment, the decision to open reduction and internal fixation was taken. A locoregional axillary nerve block was applied. A dorsal approach to the right wrist was chosen, medially to the Lister's tubercle (Figure 3), incision of the extensor retinaculum between the third and fourth compartment, radially based capsulotomy (v-shape) (Figure 4).

**Figure 5:**
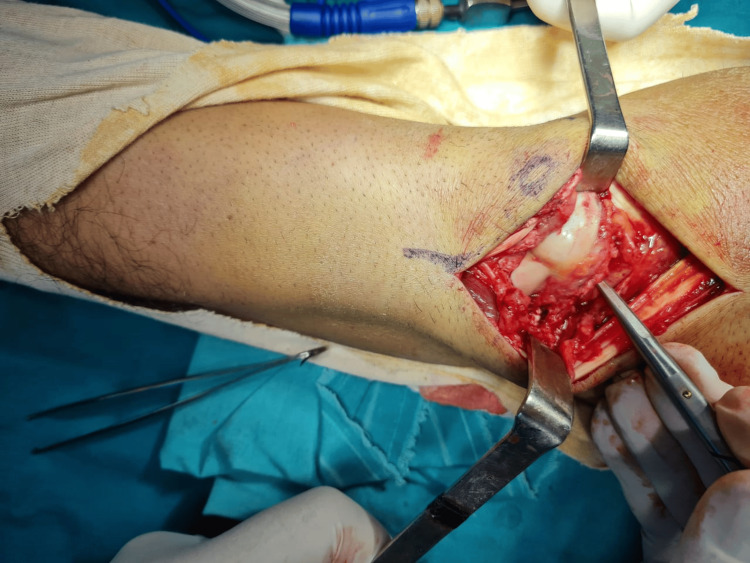
Illustration showing an intact dorsal scapholunate ligament along with the rupture of the lunotriquetral and lunocapitate ligaments

**Figure 6:**
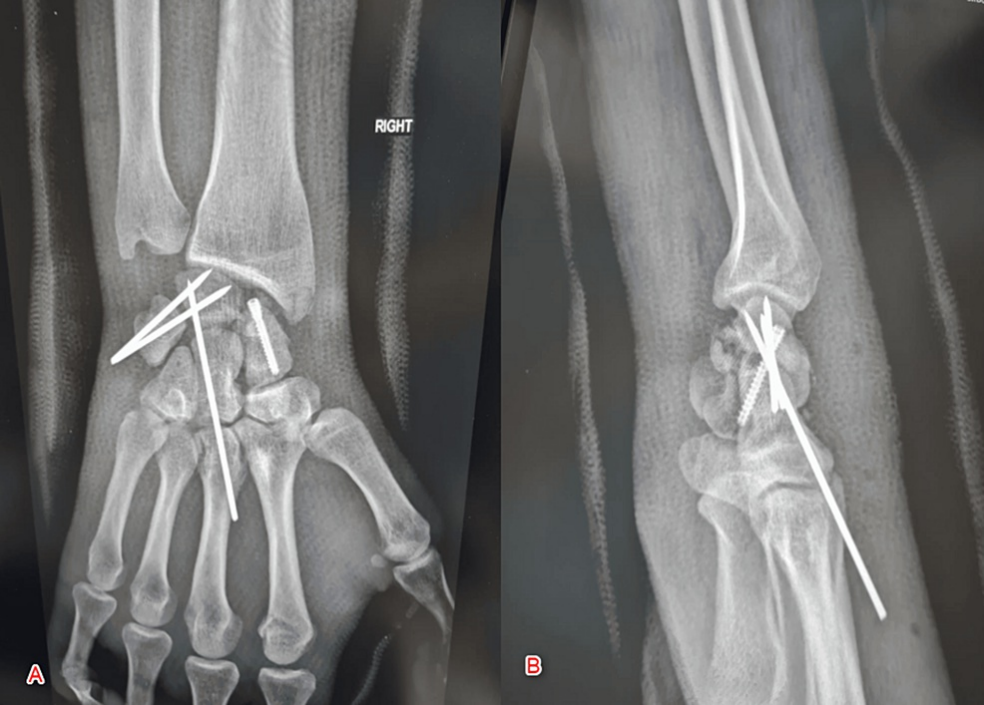
Postoperative X-ray showing stabilization of the reduction with two K-wires, reduction of the scaphoid fracture, and fixation with one screw (Herbert screw) A: Anteroposterior view; B: Lateral view

The following findings were observed: (1) normal/intact dorsal scapholunate ligament, (2) rupture of the lunotriquetral and lunocapitate ligaments (Figure 5), (3) comminution of the scaphoid (distal pole) and lunate (volar part) observed with axial traction, and (4) exaggeration of the palmar displacement. The following was then performed: (1) reduction of the triquetrum and capitate over the lunate by applying traction and dorsal force, (2) stabilization of the reduction with three K-wires pf 1.8 mm each (two from triquetrum to lunate and one from capitate to lunate) under fluoroscopic control, (3) reduction of the scaphoid fracture, and (4) fixation with one screw (Herbert screw) size 24 mm (Figure 6).

**Figure 7:**
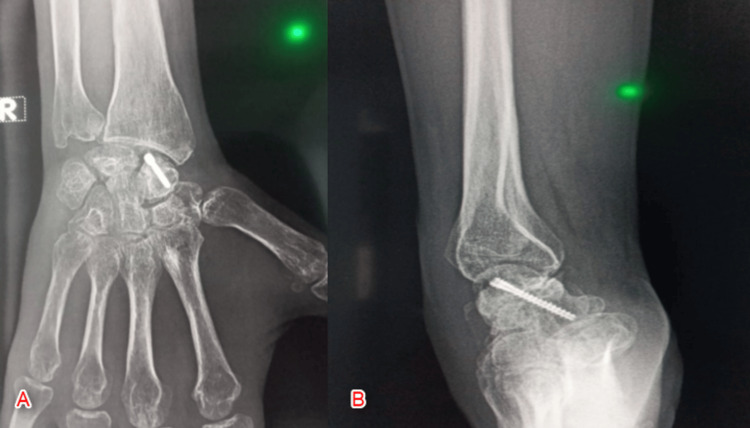
Postoperative X-ray of the right wrist at eight weeks following the removal of K wires and the cast A: Anteroposterior view; B: Lateral view

In the end, irrigation with normal 0.9% saline was done, and closure of the capsule extensor retinaculum and skin with the application of a thumb spica cast was conducted. Eight weeks after the operation, the K-wires and cast were removed, and the mobilization of the wrist commenced (Figure 7). A wrist brace was applied in the second to the eighth week, and physical exercises were initiated in the sixth week. During the final assessment follow-up of five months, the patient showed adequate mobility with no residual pain.

## Discussion

This is the first case of palmar trans-scaphoid perilunate dislocation reported in Lebanon. Based on the literature review we conducted across PubMed and Google Scholar; most similar cases were reported in Germany and France (Table 1). There is no specific age for the occurrence of this rare observation; it can occur in people from 14 to 67 years of age [4,5]. However, it is more common in young adults (20-29 years of age) [6,7]. Male predominance is observed clearly in the literature; among the 49 cases reported in the literature, only three (6%) female cases were reported [Table 1] [7]. This can be explained by the fact that middle-aged men are more commonly involved in professions that expose them to manual injuries associated with joint dislocations and/or bone fractures [8]. For instance, both hyperflexion and hyperextension of the wrist, regardless of the situation (i.e., falls) have been shown to cause this type of dislocation [9,10].

**Table 1:** Summary of reported cases of trans-scaphoid perilunar dislocation-fracture in the literature - : no data were provided. YOP: year of publication; ORIF: open reduction/internal fixation; EF: external fixation

Author (YOP)	Design	Country	Age	Sex	Injury source	Dislocation			Investigations	Management			
						Laterality	Presentation	Orientation		Intervention	Outcome	FU (months)	Complications
Fernandes (1983) [11]	Case report	-	-	-	Hyperflexion of wrist	-	-	Volar	-	Conservative	Union w/o avascular necrosis	6	-
Alt (2004) [9]	Case report	France	23	Male	Fall on outstretched hand (hyperextension)	Left	-	Dorsal	X-ray, CT, arthrography	CR	Immobilization due to prior pseudarthrosis (fusion)	2	-
Pfeiffer (1978)	Case report	Germany	-	-	-	-	-	-	-	ORIF	-	-	Arthritis, minimal loss of function and working capacity
Amar (2009) [10]	Case report	France	28	Male	Motorcycle fall	Right	Edema and deformity of the wrist + scophoid fracture	Volar	X-ray	OR + pinning	No residual pain and optimum mobility	30	-
Bahri (2000) [19]	Case series (20 patients)	France	27	Male (20/20)	-	Right (18/20); Left (2/20)	-	-	-	Reduction	Excellent results (12/20), good (6/20), and average (2/20)	3.5	-
Teklote (2000) [5]	Case report	Germany	67	Male	Electricity	Right	-	-	X-ray	ORIF	-	-	Aseptic necrosis and wrist collapse
Tan (2010) [20]	Case report	China	-	-	-	-	-	-	-	CREF	-	-	-
Gellman (1988) [18]	Case report	USA	22	Male	-	-	-	-	-	ORIF	Good functional result. Complete resolution (at 4 years)	3	Avascular necrosis
Knoll (2005) [7]	Case series (25 patients)	USA	29	Male (22/25)	-	-	-	Dorsal	Radiographs	ORIF	ROM 91%, grip strength 80%, and average extension (54o) and flexion (60o).	-	Carpal tunnel syndrome (5/25), infection (1/25).
Inoue (1997) [6]	Case series (28 patients)	Japan	29	-	-	-	-	-	-	ORIF	Satisfactory results (27/29)	24	-
Herzberg et al. (2002) [4]	Case series (14 patients)	Germany	14	-	-	-	-	Dorsal	Radiographs	ORIF: dorsal (11/14) or palmar approach (3/14)		96	None

The diagnostic follow-up of trans-scaphoid perilunate requires a thorough assessment by physical and radiographical examination. Management depends on the presenting case and possible associated factors (i.e., accompanying fracture, etc.). In some cases, conservative treatment is sufficient in the short term with good functional recovery without avascular necrosis [11]. That being said, surgery in the form of open reduction and internal fixation (ORIF) is commonly indicated in most cases. A recent study compared cases who were surgically treated to those who did not receive any form of surgical intervention. Surgically treated patients with trans-scaphoid perilunate dislocation had significantly higher mean Mayo wrist scores (88 vs. 71) with higher degrees of flexion (55° vs. 49°) and extension (54 o vs. 48o), respectively. However, the number of patients with degenerative change was slightly higher in the surgically treated group (11% vs. 9%) [12].

As closed reduction and immobilization cannot reliably maintain carpal alignment, surgical treatment is essential. In trans-scaphoid perilunar dislocation, an anatomical reduction can be achieved by closed manipulation only in 67% of cases, and more than half of patients reportedly lose the anatomic position during the first six weeks of treatment despite adequate immobilization [1]. Therefore, early surgical management is mandatory after closed reduction. Budoff suggested that surgery should be performed within the first week after injury [13]. Two months after injury, surgical reduction may not be possible, and a salvage procedure should be considered after four months. There are many types of salvage procedures such as partial wrist denervation involving combined neurectomy of the posterior interosseous nerve (PIN) and anterior interosseous nerve (AIN) through a single dorsal incision [9,14], proximal row carpectomy involving excision of the scaphoid lunate and triquetrum bones, three-corner arthrodesis involving intercarpal arthrodesis of lunate, capitate, and hamate bones with scaphoid triquetrum excision used as an autologous bone graft, four-corner arthrodesis involving intercarpal arthrodesis of the lunate, capitate, triquetrum, and hamate with scaphoid excision used as an autologous bone graft, and total wrist arthrodesis if chronic injuries with degenerative changes).

Many studies reported improved outcomes in patients who underwent open reduction internal fixation compared to those treated with closed methods. Many different open approaches to trans-scaphoid perilunar dislocation have been described, but there is still some debate regarding surgical exposure [15]. The dorsal approach allows for adequate visualization and accurate reduction of the carpus. Many surgeons recently described an arthroscopic surgical technique for trans-scaphoid perilunar dislocation, and promising short-term results have been reported including an improved postoperative range of motion with less stiffness and short rehabilitation periods [16]. In open techniques, the reported incidence of carpal arthritis following open surgery ranges from 18% to 22% within three years but increases to 50% to 100% with follow-up periods of 6 to 13 years [17]. Herzberg et al. reported the occurrence of radiocarpal or midcarpal arthritis after eight years in nearly all patients but with a poor correlation between clinical function and radiographic changes [4]. Although post-traumatic radiological arthritis is common in these cases, this does not correlate necessarily with the Mayo wrist score reflective of functional improvement [4]. Other complications associated with ORIF for trans-scaphoid perilunate dislocation include aseptic necrosis [5], avascular necrosis [18], and infection [7]. However, all of these complications are rarely encountered. Among factors that might impair wrist function after the injuries are carpal instability, scaphoid nonunion, and carpal malalignment. All of these with their consequent outcomes make it wise to choose surgery for open reduction and internal fixation.

## Conclusions

Trans-scaphoid perilunar dislocation is a high-energy injury, but the diagnosis is frequently missed. Proper history, physical exam, adequate radiological assessment, and appropriate early surgical management are important to achieve optimal outcomes. Open-reduction/internal fixation is the standard approach for this injury, although long-term follow-up data are still needed.
